# The complete genome of a baculovirus isolated from an insect of medical interest: *Lonomia obliqua* (Lepidoptera: Saturniidae)

**DOI:** 10.1038/srep23127

**Published:** 2016-06-10

**Authors:** C. W. Aragão-Silva, M. S. Andrade, D. M. P. Ardisson-Araújo, J. E. A. Fernandes, F. S. Morgado, S. N. Báo, R. H. P. Moraes, J. L. C. Wolff, F. L. Melo, B. M. Ribeiro

**Affiliations:** 1 Departamento de Biologia Celular, Instituto de Ciências Biológicas, Universidade de Brasília, Brasília, DF, Brazil; 2 Laboratório de Biologia Molecular e Virologia, Centro de Ciências Biológicas e da Saúde (CCBS), Universidade Presbiteriana Mackenzie, São Paulo, SP, Brazil; 3 Departamento de Entomologia, Instituto Butantan, Av. Vital Brasil, 1500, São Paulo, Brazil

## Abstract

*Lonomia obliqua* (Lepidoptera: Saturniidae) is a species of medical importance due to the severity of reactions caused by accidental contact with the caterpillar bristles. Several natural pathogens have been identified in *L. obliqua,* and among them the baculovirus Lonomia obliqua multiple nucleopolyhedrovirus (LoobMNPV). The complete genome of LoobMNPV was sequenced and shown to have 120,022 bp long with 134 putative open reading frames (ORFs). Phylogenetic analysis of the LoobMNPV genome showed that it belongs to *Alphabaculovirus* group I (lepidopteran-infective NPV). A total of 12 unique ORFs were identified with no homologs in other sequenced baculovirus genomes. One of these, the predicted protein encoded by *loob035,* showed significant identity to an eukaryotic transcription terminator factor (TTF2) from the Lepidoptera *Danaus plexippus,* suggesting an independent acquisition through horizontal gene transfer. Homologs of *cathepsin* and *chitinase* genes, which are involved in host integument liquefaction and viral spread, were not found in this genome. As *L. obliqua* presents a gregarious behavior during the larvae stage the impact of this deletion might be neglectable.

Although the vast majority of caterpillars species do not represent a threat to human health, members of 12 lepidopteran families may cause serious human injuries^1^. Among these, the larvae *Lonomia obliqua* (Walker, 1855) (Lepidoptera: Saturniidae) is included, causing several accidents in the Southern region of Brazil^2^. This insect has been detected feeding on plants from the families *Anacardiaceae*. and *Meliaceae* and in city orchards^2^. When in contact with the human skin, a toxin is released by the larvae bristles, resulting in reactions varying from local irritation to severe life threatening conditions such as coagulopathy, acute renal failure and hemorrhagic disorders^3^. The gregarious nature of the larvae contributes to a higher venom exposure, which aggravates the severity of the symptoms. There are indications that the occurrence of accidents with *L. obliqua* is increasing^2^, possibly due to several factors such as deforestation, the introduction of exotic plants and reduction of natural enemies^4^.

The need to control this insect population has led to the identification of several pathogens and predators, including a nematode (*Hexamermis sp*.)^5^ and a baculovirus: Lonomia obliqua multiple nucleopolyhedrovirus (LoobMNPV), which was isolated from *L. obliqua* larvae^6^. The infected larvae exhibited all the usual symptoms of a baculovirus infection, although lacking liquefaction and melanization *post mortem* phenotypes^6^. A previous study determined the sequence of a conserved baculovirus gene (*polyhedrin*) gene that showed close similarity with members of group I alphabaculovirus^6^.

The first baculovirus to be completely sequenced was the Autographa californica multiple nucleopolyhedrovirus (AcMNPV) in 1994^7^. Since then, the number of complete genomes has been growing rapidly, providing a wealth of data that contributes to the understanding of both biology and evolution of baculovirus. However, there are only three reports of complete genomes from baculoviruses isolated from species of the family Saturnidae: *Hemileuca sp. nucleopolyhedrovirus*^8^, *Philosamia cynthia nucleopolyhedrovirus*^9^, and two isolates of *Antheraea pernyi nucleopolyhedrovirus*^10^. Therefore, the complete genome of LoobMNPV provides additional data that may help the understanding of baculovirus genome evolution and adaptations to their hosts.

## Results and Discussion

### Genome Features

**Figure 1:**
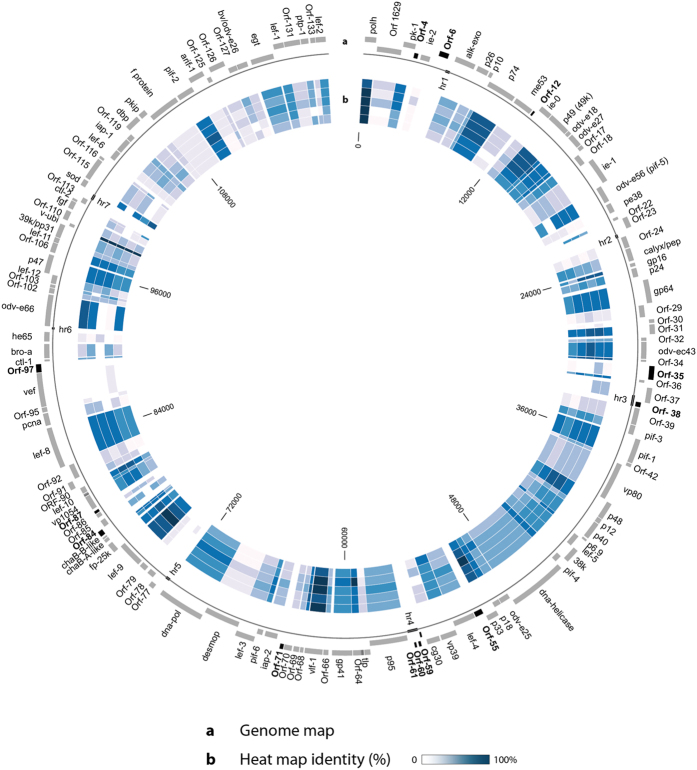
Genome map and heat map of the LoobMNPV genome. (**a**) Genome map showing all the 134 ORFs. The unique ORFs are represented in black. The outer track contains forward orientation ORFs, and the inner track contains reverse orientation ORFs. Hrs are shown on the line below the genome. (**b**) Heat map identity of the genomes of the species AcMNPV, ThorNPV, MaviMNPV, DekiNPV, and AnpeNPV (from the outside to the inside) compared to ortholog ORFs from LoobMNPV. The darker the blue, the higher is the correlated ORF identity.

The assembled genome of LoobMNPV (GenBank accession number: KP763670) is 120,023-bp long with a mean coverage of 20.5 times. We have found 134 Open Reading Frames (ORFs) coding for putative proteins with at least 50 amino acid residues (Fig. 1a and Table S1). Out of these, 120 ORFs were found in other baculoviruses and among those, three ORFs (*loob078*, *loob100* (*he65*) and *loob113*) showed higher identity with orthologs from betabaculoviruses (Table S1), confirming that horizontal transference between alphabaculovirus and betabaculovirus is indeed a common event, as previously documented^11^. Moreover, some unique ORFs were located within the *hr*3 (*loob038*) and *hr*4 (*loob060* and *loob061*). All the 37 baculovirus core genes were present, however some genes present in almost all Alpha- and Betabaculovirus^12^ genomes available were not present: Ac106/107, Ac108 and gp37.

### LoobMNPV homologous regions (*hrs*)

**Figure 2:**
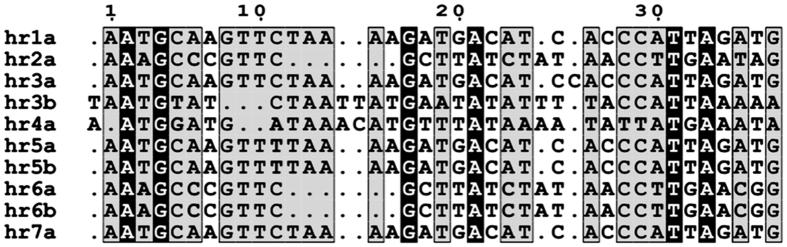
LoobMNPV *hr* palindromes. Alignment showing the position and the sequence of 38 *hr*-like imperfect palindromes found in the LoobMNPV genome, numbered sequentially, where letters designate palindromes within the same *hr*. The black shaded areas indicate residues that have strict identity, whereas grey shaded areas indicate conservation within the majority of the group (no strict identity). Dots represent gaps to achive a better alignment.

Seven homologous regions (*hrs*) were found in the LoobMNPV genome, in agreement to other baculoviruses^13^. These DNA palindromic repetitive elements are interspersed throughout most baculoviral genomes and are related to DNA replication^14^, gene transcription^15^, and possibly homologous recombination^16^. The *hr1* has 684 bp, *hr2* has 541 bp, *hr3* has 1,037 bp, *hr4* has 1,426 bp, *hr5* has 679 bp, *hr6* has 361 bp and *hr7* has 836 bp, and their size and position were confirmed by PCR (data not shown). As shown in Fig. 2, all *hrs* have various copies of a common imperfect palindromic repeat of 38 bp (*hr1a*, *hr2a*, *hr3a*, *hr3b*, *hr4a*, *hr5a*, *hr5b*, *hr6a*, *hr6b* and *hr7a*).

### LoobMNPV Phylogeny

**Figure 3:**
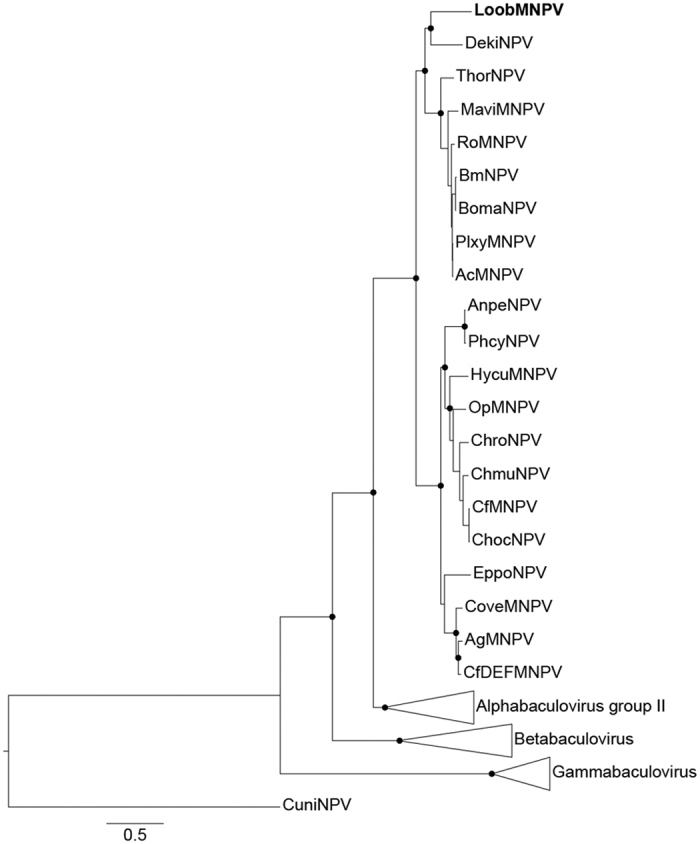
Phylogeny of baculoviruses. Phylogenetic inference of 37 conserved proteins (core genes) present in 72 baculovirus genomes from different host species. The genera *Gammabaculovirus*, *Betabaculovirus*, and group II *Alphaphabaculovirus* are collapsed. CuniNPV was used to root the tree. LoobMNPV belongs to the genus *Alphabaculovirus*, on group I, and clustered with DekiNPV. Both species are a sister clade of AcMNPV-related species.

We have performed a phylogenetic analysis (Fig. 3) based on the concatenated amino acid sequence alignment of 37 baculovirus core genes of 72 baculovirus species available in GenBank (Table S2). LoobMNPV clustered with group I alphabaculovirus. Its closest relative was found to be DekiNPV and both are basally related to AcMNPV-like viruses. This result disagrees with previous analysis based on the *polh* gene, in which LoobMNPV clustered together with both species *Amsacta albistriga nucleopolyhedrovirus* (AaNPV) and *Thysanoplusia orichalcea nucleopolyhedrovirus* (ThorNPV)^6^. However, the phylogenetic signal of highly conserved genes, such as *polh*, is usually inaccurate^17^, while phylogenetic inferences based on a large set of genes is more accurate and robust^18^.

### LoobMNPV Comparative Genomics

Based on the phylogenetic results we have selected some of the closest relatives of LoobMNPV, including AcMNPV, *Antheraea pernyi nucleopolyhedrovirus* (AnpeNPV), *Maruca vitrata nucleopolyhedrovirus* (MaviNPV), *Dendrolimus kikuchii nucleopolyhedrovirus* (DekiNPV) and ThorNPV for comparative genomics. Figure 1b and Table S1 reveal all the LoobMNPV ORFs compared to orthologs from these genomes by the level of similarity (in terms of percentage of identity and *E-values* < 10^−3^). Overall, some ORFs are more conserved (high similarity), such as the genes that correspond to *polh* (*loob0*01), *p74* (*loob010*), *p49* (*loob014*), *pif-1* (*loob041*), *vlf-1* (*loob067*), *lef-9* (*loob080*), *lef-8* (*loob093*) and *pif-2* (*loob123*); while others are more variable, presenting a lower level of similarity, but still present in almost all baculovirus genomes, such as *ORF1629* (*loob002*), *ie-2* (*loob005*), *vp80* (*loob043*), *desmoplakin* (*loob075*), *f protein* (*loob122*), *arif-1* (*loob124*) and *bv*/*odv-e26* (*loob128*).

**Figure 4:**
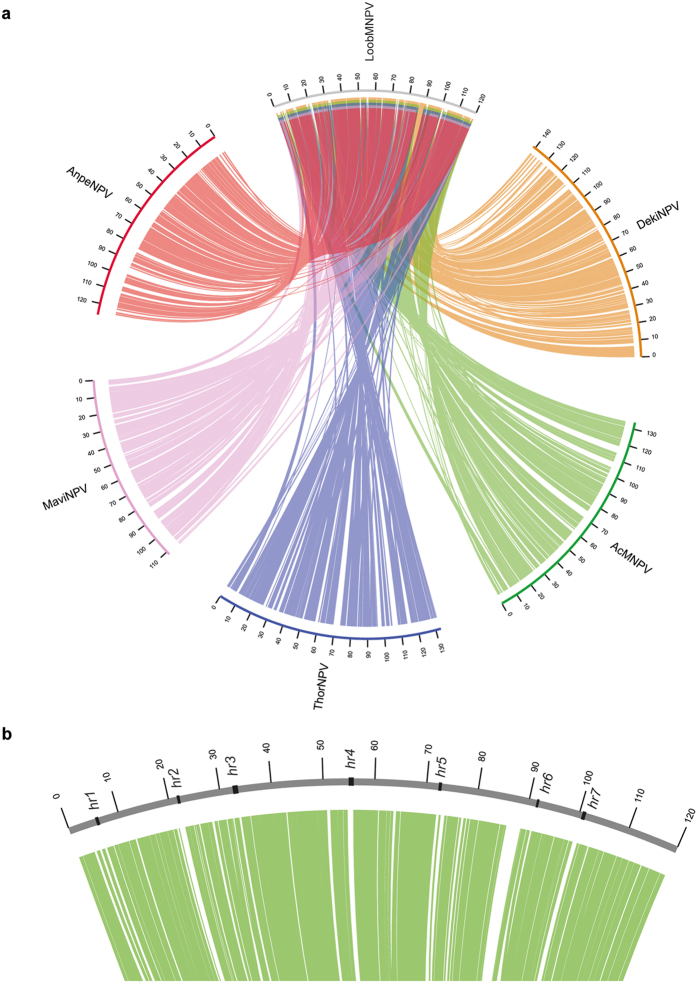
Genome sinteny of LoobMNPV. (**a**) LoobMNPV sinteny comparison to the baculoviruses AnpeNPV (salmon), MaviMNPV (light pink), ThorNPV (blue), AcMNPV (green), and DekiNPV (orange). Each line represent an ortholog ORF to LoobMNPV based on the protein identity. LoobMNPV and DekiNPV are collinear, while the other genomes show an inversion when compared to LoobMNPV. (**b**) Zoom from (**a**) on the location where all baculoviruses compared overlap, showing *hrs* located closely to LoobMNPV unique ORFs (white gaps).

Moreover, we have investigated the genome synteny among LoobMNPV and those selected alphabaculovirus genomes (Fig. 4). A circular ideogram layout shown in Fig. 4 displays lines connecting related genes, where it is possible to observe the relative position compared to LoobMNPV genome. Interestingly, we have observed that LoobMNPV and DekiNPV show high collinearity, and both present genome inversions and rearrangement in contrast to the genomes of AcMNPV, AnpeNPV, MaviNPV, and ThorNPV. The inverted regions were adjacent or flanked by the *hr1* and *hr2* (Fig. S1). These findings support the phylogenetic results presented in Fig. 3 and suggests that the inversions were originated in a common ancestor of both LoobMNPV and DekiNPV. These inversions are an autapomorphy when compared to the AcMNPV-like viruses. Previous studies have shown that rearrangements in the baculovirus genomes reflect evolutionary history, with more closely related viruses presenting higher genome collinearity^19^.

Furthermore, when analyzing the region where all genomes overlap with LoobMNPV (Fig. 4b), we have observed that unique ORFs in LoobMNPV (gaps with no correspondence to the other genomes) are interestingly found near *hrs*. According to previous reports, gene rearrangements and acquisitions are of common occurrence on proximities of *hrs*^20^, confirming the possibility of gene transfers to viruses by homologous recombination^21^, which could be also facilitated by factors such as the prevalence of various pathogens infecting the same host^22^, as well as concomitant infections in field populations^23^.

### LoobMNPV unique ORFs

LoobMNPV genome showed 12 genes that do not have any match among baculoviruses. These ORFs are *loob004*, *loob006*, *loob012*, *loob035*, *loob038*, *loob055*, *loob059*, *loob060*, *loob061*, *loob071*, *loob084*, and *loob097*. There are three possible mechanisms for gene acquisition: extensive sequence divergence, which could push homolog genes below the similarity threshold; gene recombination, which produces novel protein products; and horizontal gene transfer (HGT). This third possibility is expected to be detectable by gene similarity from phylogenetically distinct species^24^. For each of these LoobMNPV unique ORFs, we have searched for baculovirus promoter motifs within 200 bp upstream of the start codon. The late promoter motif TAAG, that appears to be necessary for late transcription by the viral RNA polymerase^25^ was found in *loob004*, *loob012*, *loob030*, *loob038*, *loob055*, and *loob071*. However, further experiments are necessary to confirm whether these ORFs encode *bona fide* proteins. Moreover, these novel ORFs were searched for known domains and eight of theses ORFs (*loob004*, *loob006*, *loob012*, *loob038*, *loob055*, *loob061*, *loob071*, and *loob097*) did not match any predicted domain. The remaining will be discussed below.

### Insect immune system-associated domain

The *loob060* has an immunoglobulin-like domain, which has been found in some insect proteins, such as the *hemolin*, an hemolymph component that plays a role in bacterial surface binding, forming a protein complex that initiates the immune response^26^. *Hemolin* has also been found in the transcripts of *L. obliqua* bristles^2^. Several immunomodulators encoded by viruses have been described^27^ and may be involved in regulating the immune system and protecting virus-infected cells from the attack of other cells from the immune system^28^,^29^. For viruses, the expression of these proteins may indicate beneficial susceptibility in multiple pathogen infections, by protecting the host against opportunistic pathogens, reducing competition and benefiting viral propagation^29^. For instance, other saturniid-related alphabaculovirus species HespNPV expresses a functional insect-related serine protease inhibitor (serpin) in its genome that is likely related to host immunity modulation and virulence^8^,^28^.

### Transcription factor-related domain (*loob035*)

One unique ORF demonstrated high correspondence to the eukaryotic transcription terminator factor type 2 (TTF2) from the butterfly *Danaus plexippus* (GenBank: EHJ68439), with 44% pairwise identity and *E-value* equals to 3e10^−11^. However, when filtering this result, in order to focus only on the family *Baculoviridae*, the referred gene presented higher similarity to the Global Transactivator (GTA) gene from the AnpeNPV (YP_611073), with 66% of identity and an *E-value* of 1e10^−6^.

Transcription Factors (TFs) in general are fundamental in a broad array of any cellular processes due to its ability of causing changes in downstream gene expression patterns^30^. GTA genes are observed in members from the group I alphabaculovirus. According to a previous study^31^, baculovirus GTAs play an important role in transcriptional activation of viral genes and were probably originated by HGT from the host to the common ancestor of the clade that includes AcMNPV, Bombyx mori nucleopolyhedrovirus (BmNPV), Orgyia pseudotsugata multicapsid nucleopolyhedrovirus (OpMNPV), and Epiphyas postvittana nucleopolyhedrovirus (EppoNPV). Katsuma, *et al.*^32^ found that a GTA homolog from BmNPV acts as a viral virulence factor in insect larvae, and may be required for activation of host and/or viral genes, increasing the speed of host killing. Based on domain analysis, we have found that the *loob035*, TTF2, and GTA genes are members of the SNF2 family. This family of genes encodes proteins with sequence motifs similar to those found in many DNA and RNA helicase protein families, and also proteins from a variety of species with roles in cellular processes such as transcriptional regulation, DNA recombination, chromatin unwinding and various other types of DNA repair^33^. In this regard, the possible acquisition of *loob035* might be involved in the inhibition of the host transcriptional machinery in order to benefit viral expression.

**Figure 5:**
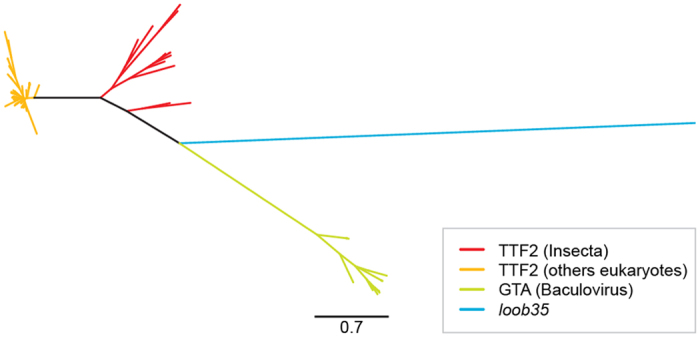
Phylogeny of GTAs and TTF2 genes. Unrooted maximum likelihood phylogeny of the data set containing genes that correspond to TTF2 from Insecta (red), TTF2 from other eukaryote (orange), GTA from group I *Alphabaculovirus* (green), and *loob035* (blue).

To analyze whether *loob035* has been independently acquired from the host insect through HGT, or is a divergent baculovirus GTA gene, a phylogenetic analysis has been performeddone, based on an amino acid alignment containing both TTF2 and GTA sequences. As shown in Fig. 5, *loob035* presented a long branch size, which indicates great divergence from the other sequences, possibly due to positive selection^34^. Actually, TFs are overrepresented among genes predicted to be positively selected in previous genome-wide selection studies^35^. To confirm whether *loob035* is in fact a new acquisition from insect host or a divergent baculovirus GTA gene, we compared the likelihood of a tree constraining *loob035* to the GTA alphabaculovirus group (lnL = −14940.61) with the likelihood of a tree constraining *loob035* into TTF2 group (lnL = −1455.06). We argue that the likelihood differences in combination with high non-parametric bootstrap values and high posterior probabilities constitute considerable evidence that *loob035* clusters with the group of TTF2 genes, corroborating to the possibility of a novel HGT.

**Figure 6:**
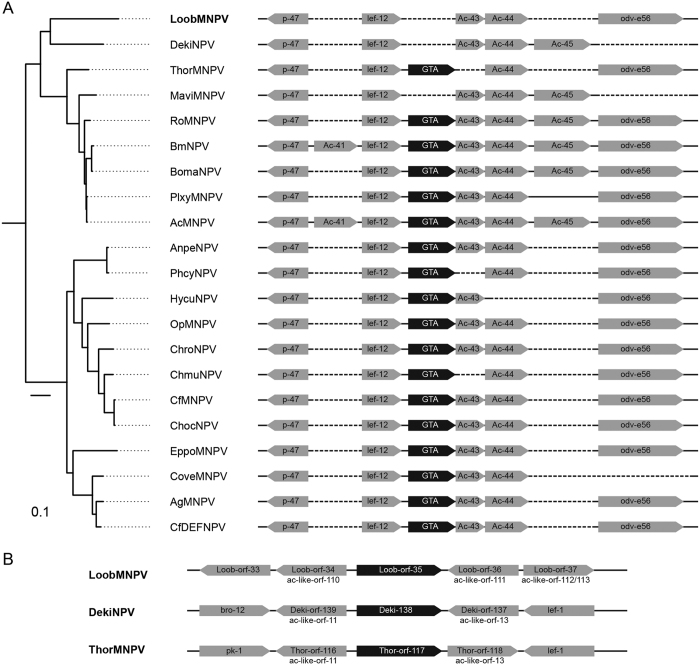
GTA genomic context in several alphabaculoviruses. (**a**) The GTA gene is present in almost all group I *Alphabaculovirus* and is shown here aligned to the phylogeny previously showed on Fig. 3. In AcMNPV, AgMNPV, AnpeNPV, BmNPV, BomaNPV, CfMNPV, ChocNPV, ChmuNPV, ChroNPV, DekiNPV, EppoNPV, HycuMNPV, LoobMNPV, MaviMNPV, OpMNPV, PhcyNPV, PlxyMNPV, RoMNPV e ThorNPV, the GTA gene is always in between *p-47* (followed by *lef-12*) and *odv-e56.* LoobMNPV, DekiNPV and MaviMNPV lack the GTA gene. Dotted lines represent absence of ORFs. (**b**) The position of *loob035* in the genome of LoobMNPV is located between homologs of both *ac110*-like (*loob034*) and *ac111*-like (*loob036*). The position of two *loob035* homologs (Table S1) from DekiNPV_Orf138 and ThorNPV_Orf117 are also represented in different genome contexts.

We further confirmed this independent acquisition hypothesis by analyzing the genomic context of baculovirus GTA genes. As shown in Fig. 6a, GTA genes are encountered in all group I alphabaculoviruses:AcMNPV, Anticarsia gemmatalis nucleopolyhedrovirus (AgMNPV), AnpeNPV, BmNPV, Bombyx mandarina nucleopolyhedrovirus (BomaNPV), Choristoneura fumiferana multicapsid nucleopolyhedrovirus (CfMNPV), Choristoneura occidentalis nucleopolyhedrovirus (ChocNPV), Choristoneura murinana nucleopolyhedrovirus (ChmuNPV), Choristoneura nucleopolyhedrovirus roaceana (ChroNPV), EppoNPV, Hyphantria cunea nucleopolyhedrovirus (HycuMNPV), Orgyia pseudotsugata multicapsid nucleopolyhedrovirus (OpMNPV), Philosamia cynthia nucleopolyhedrovirus (PhcyNPV), Plutella xylostella multiple nucleopolyhedrovirus (PlxyMNPV), Rachiplusia ou multicapsid nucleopolyhedrovirus (RoMNPV) and ThorNPV; except in MaviMNPV, LoobMNPV and DekiNPV, within a conserved position between *lef-12* and *odv-e66*. However, in LoobMNPV, the gene located in this position is *loob102*, that corresponds to an AcMNPV-like gene (*ac044*). On the other hand, *loob035* is inserted in a completely different genome context, located between both the ac110- and the *ac*111-like genes (Fig. 6b), confirming that *loob035* has probably a different origin not related to the GTA gene. Besides, *loob035* homologs found in DekiNPV (Orf 138) and in ThorNPV (Orf 117), according to Table S1, are also inserted in a different context (Fig. 6b).

Notably, *loob035* diverges greatly from all the other compared sequences (Fig. S2), showing that these sequences present similarity only because they all contain the SNF2 conserved domain. Hughes & Friedman^31^ found that SNF2 baculovirus gene family has homologs in cellular organisms, and clustered closer to homologs in insects (*Drosophila)*, according to the reconstruction of the evolutionary relationship among genes that were potentially acquired through HGT in comparison to baculovirus phylogeny.

#### The absence of *cathepsin* and *chitinase* genes in LoobMNPV

Interestingly, LoobMNPV does not encode two common baculovirus genes that are responsible for the *post mortem* host melanization and liquefaction benefiting virus dissemination: the enzymes *cathepsin* (*v-cath*) and *chitinase* (*chiA*) genes^36^. Besides the involvement of these genes in the horizontal spread of the virus in the field^37^, it has been reported that the *chiA* gene and the pro-form of *v-cath* interact directly and are dependent on each other for the promotion of host liquefaction, and therefore, they are usually acquired or lost together^38^, since they are adjacent genes on the genome. Among alphabaculovirus from group I, only LoobMNPV, AgMNPV and PhcyNPV lack *v-cath* and *chiA* genes. The recombinant introduction of *v-cath* and *chiA* genes from *Choristoneura fumiferana DEF multiple nucleopolyhedrovirus* (CfDEFNPV) into AgMNPV genome improves production of occlusion bodies and insecticidal activity during A*. gemmatalis* larvae infection^39^.

Several hosts from the family Saturniidae^40^, as well as from Noctuidae, present gregarious behavior^41^, which may facilitate virus dispersion to new susceptible individuals and reduce selective pressure for the maintenance of *v-cath* and *chiA* genes, as observed for LoobMNPV, AgMNPV, and PhcyNPV. However, HespNPV and AnpeNPV also infect gregarious hosts from Saturniidae family, but have *v-cath* and *chiA* genes in their genomes. Therefore, it remains to be determined whether the host behavior it is indeed a selective factor for *cath* and *chiA* genes maintenance.

### Conclusions

In this work, we described the first complete genome sequence of a baculovirus isolated from a species of medical interest. LoobMNPV is located on a basal position of group I alphabaculovirus and presents inversions in large proportions when compared to the other related genomes. During evolution, fluxes in the genomes content, such as genes acquisitions and losses, pressured by positive selection, could possibly implicate in shifts on the evolutionary dynamics, by the occurrence of events of adaptation. Therefore, the elucidation of novel genomes will help the studies on baculovirus evolution, proportioning deeper knowledge and understanding of baculovirus as a whole group.

## Materials and Methods

### Virus origin

The LoobMNPV used in this work was collected in the year 2000, from orchards in the state of Santa Catarina, Brazil^6^.

### Virus purification, DNA extraction and sequencing

Occlusion bodies (OBs) from dead *L. obliqua* larvae were purified and dissolved in an alkaline solution for DNA purification, all according to previously published protocol to O’Reilly *et al.*^42^. The quantity and quality of the isolated DNA was determined by electrophoresis on 0.8% agarose gel (data not shown). The DNA was submitted to genome sequencing throughout the pyrosequencing technique performed by the 454 Genome Sequencer (GS) FLX™ Standard (Roche) at Macrogen Inc (Seoul, Republic of Korea).

### Genome assembly and annotation

Genome *de novo* assembly and annotation was performed using Geneious version 7.1.6 (available at http://www.geneious.com/)^43^ considering the following established parameters to select the Open Reading Frames (ORFs): start codons corresponding to the methionine residue (ATG), minimal overlapping of adjacent ORFs, and ORFs as large as 150 bp. No gaps were found between connected contigs, only a 700 bp region of low coverage, that was confirmed by PCR, In order to validate assembly, an *in silico* digestion was carried out using *Bam*HI, *Eco*RI and *Pst*I restriction enzymes and comparing to a previously published restriction profile^6^, that used the same virus isolate as the one used in this work (Table S3). ORFs were annotated using BLASTx and PSI-BLASTp searches against the NCBI non-redundant protein database^44^. When compared to all baculoviruses genomes available at Genbank, unique ORFs were considered when no significant database hits (*E-values* > 10^−3^) were found, and for those, a more sensitive search was performed using HMMer-search against the PFAM-database^45^. The presence of protein specific domains in HMMer-search unique ORFs was investigated using InterProScan database^46^. The regions within 200 bp upstream of the putative unique ORFs were screened for the presence of TATA-box and CAGT^47^, as well as GATA motifs^48^.

### Homologous regions (*hr*s)

The homologous regions (*hrs*) were annotated using DOTPLOT analysis and Tandem Repeat Finder (http://tandem.bu.edu/trf/trf.html)^49^. An alignment of the repeat unit of each *hr* was performed using the MAFFT method^50^. To confirm the size and position of the *hr*s among the genome, PCRs were performed, using the isolated LoobMNPV DNA and the following primers: *hr1* (F: AGA GTT GGA AAT TTC GCG CTC and R: GTT TTT ACT CTG TCC GCG CG); *hr2* (F: CCC GCT AAT GAA CCG TGT GA and R: AAC CGT TTA AAT CCT TCG TGT); *hr3* (F: GCT GGA GTA AAT TGT TCA ATC GC and R: TTT CCA TAA CGG GGT GCC AA); *hr4* (F: TAG GGC ACA ATA GCA GCA GC and R: ACG TGC CAA GTC GAA TCT GA); *hr5* (F: CGC ATA ACC TTT AGC GTG ACT and R: CTG AAA CGC GAC AAC AGT CC); *hr6* (F: AGA GTT GGA AAT TTC GCG CTC and R: TCA TGT CGG CCA ATG AGG AC) and *hr7* (F: AAT GCG CAA AAG AAC GGG TC and R: AAC AAC TAA ACT GCG CCC CA).

### Phylogenetic analysis and genome comparisons

A MAFFT alignment was performed using amino acid sequences from the predicted 37 core genes from 72 baculovirus genomes (one isolate from each baculovirus species) available in the GenBank up to the date. The phylogeny was inferred by three different phylogenetic algorithms including FastTree^51^, which uses SH-like method for branch support, along with RAxML^52^ and PhyML^53^ that use the bootstrap method for branch support. The phylogenies were inferred by the maximum likelihood (ML) method, along with GTR model of DNA evolution, estimated with JmodelProttest 2.4 software program^54^. Moreover, a genomic comparison was performed using blastp identity results of a protein dataset with four alphabaculovirus genomes AcMNPV, AnpeNPV, MaviNPV, DekiNPV and ThorNPV. This dataset was compared using CGView Comparison Tool^55^ and the results were plotted using CIRCOS^56^. Likewise, to investigate the evolutionary relationship between *loob035* and the GTA and TTF2 genes, a MAFFT alignment was performed using 14 GTA genes from alphabaculoviruses and 62 TTF2 genes from insects and other eukaryotes. This phylogenetic tree was done by ML method implemented in PhyML^53^, with JTT substitution model of amino acid evolution, and 100 repetitions of a non-parametric bootstrap. A multiple alignment was performed on the T-coffee platform^57^ with the predicted *loob035* along with the GTA and TTF2 proteins, and was submitted to the ESPript 3 platform^58^. The two most similar crystal structures to *loob035* were used to predict the secondary structures of the alignment. They were extracted from the Protein Data Bank (PDB ID 3mwy^59^ and PDB ID 1z63^60^), and were aligned along with the GTA genes from AnpeNPV and EppoMPV and TTF2 genes from *Danaus plexippus, Bombyx mori, Chelonia mydas* and *Pterotopus alecto*. To show statistical evidence concerning the best hypothesis for *loob035* position in the GTA/TTF2 tree, the Bayes factor estimator^61^ from MrBayes program^62^ was used.

## Additional Information

**How to cite this article**: Aragão-Silva, C. W. *et al.* The complete genome of a baculovirus isolated from an insect of medical interest: *Lonomia obliqua* (Lepidoptera: Saturniidae). *Sci. Rep.* **6**, 23127; doi: 10.1038/srep23127 (2016).

## Supplementary Material

Supplementary Information
