# Supplementary material for: The complete genome of a baculovirus isolated from an insect of medical interest: Lonomia obliqua (Lepidoptera: Saturniidae)
Source: Sci Rep. 2016 Jun 10;6:23127. doi: 10.1038/srep23127 (PMC4901303; doi:10.1038/srep23127)
Supplement: Supplementary Information [file srep23127-s1.pdf]

The complete genome of a baculovirus isolated from an insect of medical interest: *Lonomia obliqua* (Lepidoptera: Saturniidae)

Aragão-Silva, C.W.[1]; Andrade, M. S.[1];Ardisson-Araújo, D.M.P. [1];Fernandes J.E.A.[1]; Morgado, F. S.[1]; Báo, S.N. [1] Moraes, R. H. P.[3]; Wolff, J. L. C.[2]; Melo, F.L[1]; Ribeiro, B.M.[1]*

[1]Departamento de Biologia Celular, Instituto de Ciências Biológicas, Universidade de Brasília, Brasília, DF, Brazil; [2]Laboratório de Biologia Molecular e Virologia, Centro de Ciências Biológicas e da Saúde (CCBS), Universidade Presbiteriana Mackenzie, São Paulo, SP, Brazil; [3]Departamento de Entomologia, Instituto Butantan, Av. Vital Brasil, 1500, São Paulo, Brazil.

*Corresponding author:

Bergmann M. Ribeiro

Laboratory of Baculovirus

Cell Biology Department

University of Brasilia

Brasilia, DF, 70910-900

tel: 55-61-3107-0478

email: bergmann@unb.br

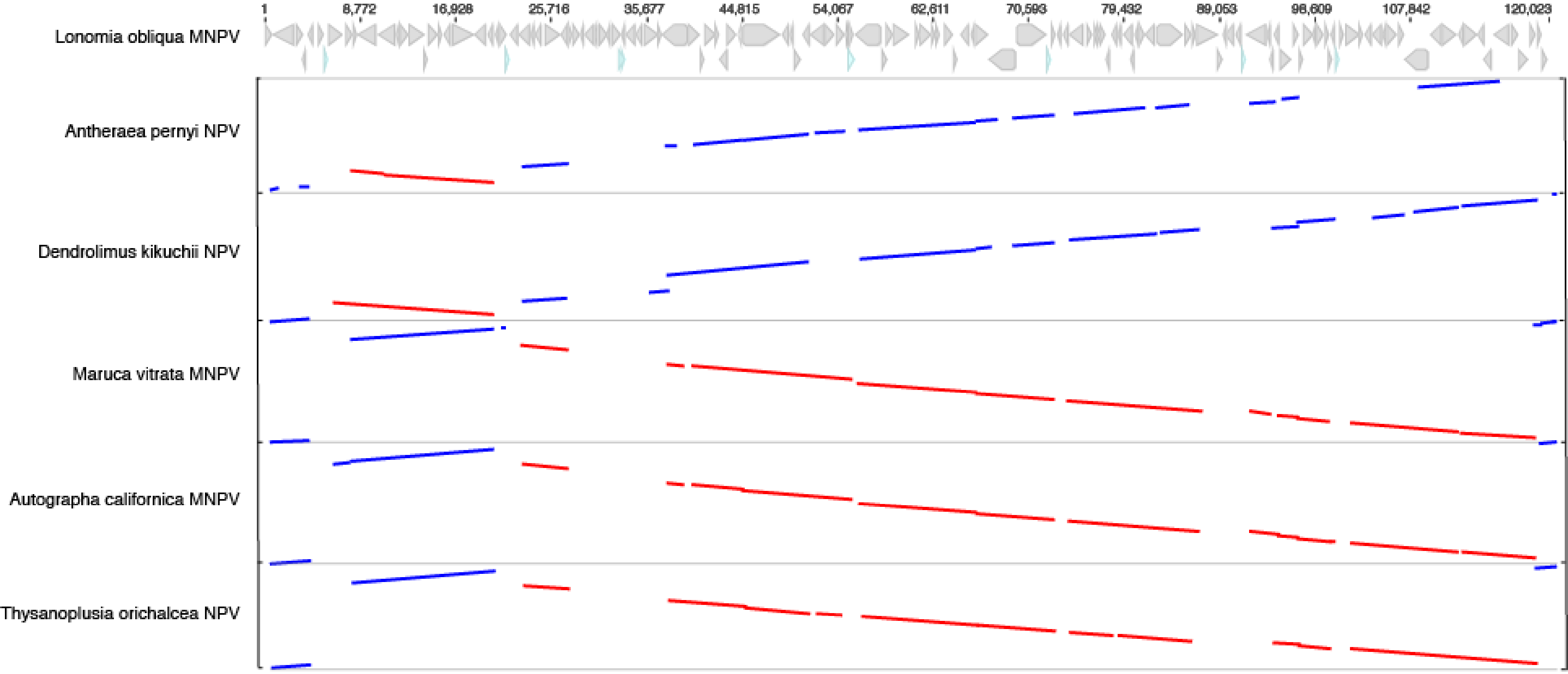

Figure S1. Pairwise alignment of LoobMNPV genomic DNA sequence to AcMNPV, ThorNPV, MaviMNPV, DekiNPV, and AnpeNPV, inverted regions are showed in red. The dot plot was generated using LASTZ (version 1.02).

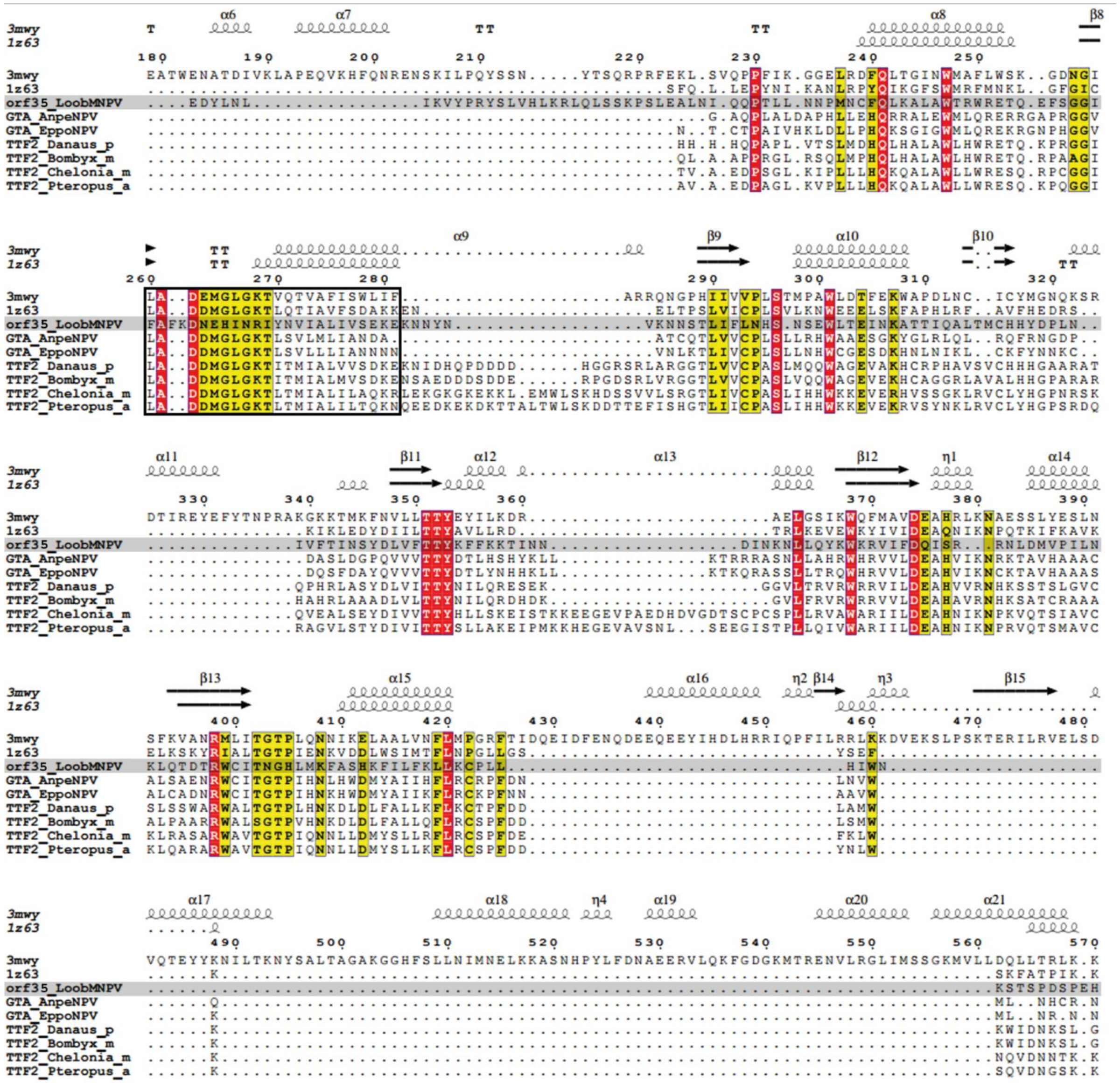

Figure S2. *loob035* multiple alignment. Secondary structures of *loob035* along with its two most similar crystal structures (3mwy and z63), GTA genes from AnpeNPV and EppoNPV, and TTF2 genes from *Danaus plexippus, Bombyx mori, Chelonia mydas*, and *Pteropus alecto.* The black box highlights a part of the alignment that clearly shows how *loob035* greatly diverges from all the other sequences in the alignment. Strictly conserved residues are boxed in a red background with white character, and highly conserved residues with similarity across groups are boxed in a yellow background with black character. The secondary structure elements of SNF2 conserved domain are shown above the sequences, and every ten residues are indicated with a dot. Protein secondary structure elements such as α-helices (coils) and β-strands (arrows) are also shown.

Table S1. The positions, orientations and size of the 134 putative ORFs identified in the LoobMNPV genome in comparison with the homologous ORFs from the baculovirus AcMNPV, AnpeNPV, MaviMNPV and DekiNPV, showing percentage amino acid (aa) sequence identity.

				AcMNPV		AnpeNPV		MaviMNPV		DekiNPV		ThorNPV	
**LoobNPV ORFs**	**Name**	**Position (nt)**	**Lenght (aa)**	**ORF**	**Ident %**	**ORF**	**Ident %**	**ORF**	**Ident %**	**ORF**	**Ident %**	**ORF**	**Ident %**
1	polh	1→750	250	Ac-PH	84,02	polyhedrin	91,8	polh	90,57	polh	91,36	ph	90,57
2	ORF 1629	823←2454	544	Ac-ORF1629	45,3	1629-capsid	26,55	orf1629	45,3			pp78/83	36,71
3	pk-1	2465→3274	269	pk-1	71,43	pk-1	58,96	pk-1	71,64	pk-1	62,69	pk-1	70,57
4	*loob004*	3271←3573	101										
5	ie-2	3728←4375	215	pe-38	30,61	ie-2	25	ie-2	20,99	ie-2	26,53	ie-2	40
6	*loob006*	4812→5456	214										
7	alk-exo	5893→7239	449	alk-exo	55,79	alk-exo	45,21	alk-exo	53,77	alk-exo	52,55	alk-exo	53,88
8	p26	7392→8168	258	Ac-p26	54,1	p26	45,7	p26	54,1	p26	46,22	p26	48,94
9	p10	8227→8487	87	Ac-p10	58,57	p10	60,81	p10	59,15	p10	61,43	p10	57,75
10	p74	8488←10437	650	p74	82,69	p74	75,27	p74	78,05	p74	76,31	p74	81,76
11	me 53	10540←11835	432	me53	44,98	me 53	36,27	me53	44,06			me53	41,15
12	*Loob012*	11893←12063	57										
13	ie-0	12149→12967	272	ie-0	46,44	ie-0	47,35	ie-0	46,44	ie-0	47,1	ie-0	45,26
14	p49(49k)	12980→14422	480	p49(49k)	82,77	p49(49k)	68,41	p49(49k)	81,7	me53	70,37	p49(49k)	82,77
15	odv-e18	14419→14691	91	odv-e18	75,86	odv-e18	62,5	odv-e18	72,53	odv-e18	63,04	odv-e18	79,37
16	odv-e27	14738→15599	286	odv-e27	72,6	odv-e27	62,02	odv-e27	72,76	odv-ec27	65,17	odv-ec27	72,82
17	*loob017*	15613→15897	95	AcOrf-145	74,03	Anpe-ORF136	65	Mv-ORF115	73,81	Deki-ORF10	75	Thor-ORF136	71,43
18	*loob018*	15969←16682	237	AcOrf-146	67,96	Anpe-ORF137	47,57	Mv-ORF114	65,05	Deki-ORF9	58,45	Thor-ORF137	63,9
19	ie-1	16738→18405	556	ie-1	46,26	ie-1	35,74	ie-1	47,12	ie-1	50,21	ie-1	45,56
20	odve-56	18463←19590	376	odve-56	72,7	odve-56	67,29	odv-e56	68,65			odv-e56	73,56
21	pe38	19719←20363	214	pe38	28,42	pe38	33,64	pe38	29,73	pe38	34,81	pe38	28,49
22	*loob022*	20706←20921	71	AcOrf-152	60,47			Mv-ORF120	65,12			Thor-ORF143	64,29
23	*loob023**	20958→21680	240										

24	*Loob0*24	22071←22862	263	AcOrf-132	35,62	Anpe-ORF123	33,79	Mv-ORF101	33,94	Deki-ORF20	28,68	Thor-ORF125	29,75
25	calyx/pep	22895←23765	289	calyx/pep	52,57	calyx/pep	67,47	calyx/pep	52,01	calyx/pep	67,46	calyx/pep	56,35
26	gp16	23887←24147	87	Ac-gp16	80,49	gp16	64,63	gp16	81,71	gp16	50	gp16	79,27
27	p24	24240←24854	204	p24	67,54	p24	55,73	p24	60,94	p24	62,24	p24	59,39
28	gp64	25121→26653	510	gp64	72,92	gp64	67,85	gp64	73,82	gp64	73,32	gp64	73,76
29	*loob0*29	26702←27514	270	AcOrf-124	36,4	Anpe-ORF114	25	Mv-ORF93	37,39	Deki-ORF35	35,71	Thor-ORF117	36,84
30	*loob0*30	27660→27926	88							Deki-ORF33	33,82	Thor-ORF117	36,76
31	*loob0*31	27986→28634	215	AcOrf-106	78,57	Anpe-ORF99	71,71	Mv-ORF83	79,23	Deki-ORF47	75	Thor-ORF101	79,72
32	*loob0*32	28810←29062	84	AcOrf-108	55,56	Anpe-ORF100	46,15	Mv-ORF84	57,41	Deki-ORF46	52,73	Thor-ORF102	61,11
33	odv-ec43	29084←30229	381	AcOrf-109	75,7	Anpe-ORF101	63,17	Mv-ORF85	76,55	Deki-ORF45	71,68	Thor-ORF103	74,94
34	*loob0*34	30258←30425	55	AcOrf-110	81,48	Anpe-ORF103	74,07	Mv-ORF86	79,63	Deki-ORF49	83,64	Thor-ORF104	79,63
35	*loob0*35	30630→31538	302	Ac-GTA	28,11	global transactivator	24,65			Deki-ORF138	46,88	Thor-ORF117	50
36	*loob0*36	31581←31784	67	AcOrf-111	73,13	Anpe-ORF104	56,92	Mv-ORF87	65,62	Deki-ORF42	74,63	Thor-ORF105	74,63
37	*loob0*37	32024→33028	334	AcOrf-113	47,93							Thor-ORF106	53,23
38	*loob0*38	33038←33391	117										
39	*loob0*39	33445←34579	378	AcOrf-114	40,48	Anpe-ORF106	33,97	Mv-ORF88	38,13	Deki-ORF40	30,95	Thor-ORF109	40,94
40	pif-3	34585←35238	218	AcOrf-115	62,63	Anpe-ORF107	63,19	pif-3	63,16	pif-3	58,03	pif-3	63,16
41	pif-1	35367→36968	533	AcOrf-119	74,67	pif-1	70,16	pif-1	73,5	pif-1	67,89	pif-1	73,92
42	*loob0*42	36984→37235	83	AcOrf-120	53,75	Anpe-ORF112	32,5	Mv-ORF92	53,09	Deki-ORF32	50	Thor-ORF114	55
43	vp80	37262←39775	837	vp80	50,99	p87	52,47	vp80	47,23	p87	47,22	vp80	49,02
44	p48	39802→40974	391	p48	78,72	p48	66,92	p45	76,92	p45	74,68	p48	78,52

45	p12	40955→41311	118	AcOrf-102	59,04	p12	48,31	p12	54,26	p12	44,35	p12	50,83
46	p40	41317→42456	380	p40	67,02	p40	52,53	p40	65,96	p40	61,92	bv/odv-c42	66,14
47	p6.9	42521→42694	57	p6.9	79,31	p6.9	79,31	p6.9	42,85	p6.9	41,09	p6.9	77,58
48	lef- 5	42691←43500	270	lef- 5	65,04	lef- 5	53,05	lef-5	64,79	lef-5	62,18	lef-5	63,91
49	38k	43435→44391	319	38k	65,09	38k	54,84	38k	63,84	38k	56,83	38k	63,86
50	pif-4	44413←44922	170	AcOrf-96	76,92	Anpe-ORF88	69,28	19kDa protein	69,05	odv-e28	73,49	pif-4	75,9
51	DNA helicase	44924→48642	1,239	DNA helicase	59,86	DNA helicase	50,72	DNA helicase	58,24	DNA helicase	61,97	DNA helicase	59,58
52	odv e-25	48673←49389	229	odv e-25	55,17	odv e-25	57,14	odv e-25	55,6	odv e-25	73,68	odv e-25	59,48
53	p18	49370←49861	163	AcOrf-93	75,46	p18	65,62	Mv-ORF70	74,38	p18	77,3	p18	74,69
54	p33	49860→50621	253	AcOrf-92	84,11	p33	73,52	p33	83,53	p33	78,93	Sox	84,11
55	*loob0*55	50660←51235	191										
56	lef- 4	51257←52723	488	Ac-lef4	60,21	lef- 4	48,77	lef-4	60,21	lef-4	55,44	lef-4	57,32
57	vp39	52750→53802	351	vp39	63,71	vp39	70,48	vp39	64,81	vp39	69,82	vp39	67,09
58	cg30	53869→54729	287	cg30	44,13	cg30	32,53			cg30	49,81	cg30	46,26
59	*loob0*59	54907←55065	53										
60	*loob0*60	55080→55277	65										
61	*loob0*61	55374→55547	57										
62	p95	55871←58378	835	p95	61,9	vp91	54,33	p95	59,84	vp91/p9	56,58	vp91	60,54
63	Tlp	58347→58949	200	Ac-TLP	40	Telokin	24,63	tlp20	38,12	Deki-ORF72	38,61	tlp	43,08
64	*loob0*64	58765→59487	241	AcOrf-81	75,58	Anpe-ORF77	69,31	Mv-ORF64	72,84	Deki-ORf73	89,71	Thor-ORF76	75,22
65	gp41	59493→60818	441	gp41	64,83	gp41	64,82	gp41	63,64	gp41	73,75	gp41	64,46
66	*loob0*66	61010→61367	118	AcOrf-78	59,46	Anpe-ORF74	52,83	Mv-RF61	60,71	Deki-ORF76	51,22	Thor-ORF73	57,66
67	vlf-1	61385→62554	389	vlf-1	89,94	vlf-1	80,18	vlf-1	87,64	vlf-1	86,78	vlf-1	82,17
68	*loob0*68	62603→62857	84	AcOrf-76	78,57	Anpe-ORF72	73,81	Mv-ORF59	76,19	Deki-ORF78	75,29	Thor-ORF71	78,57
69	*loob0*69	62875→63276	134	AcOrf-75	53,38	Anpe-ORF70	43,85	Mv-ORF58	53,38	Deki-ORF79	53,38	Thor-ORF106	51,88
70	*loob0*70	63344→63886	180	AcOrf-74	46,67	Anpe-ORF69	44,58	Mv-ORF57	46,43	Deki-ORF80	49,12	Thor-ORF70	46,06
71	*loob0*71	63886→64155	90										
72	iap-2	64413←65264	283	iap-2	53,31	iap-2	41,28	iap-2	54,41	iap-2	48,35	iap-2	54,41
73	pif-6	65392←65796	135	AcOrf -68	70,69	Anpe-ORF64	67,35	Mv-ORF52	69,83	Deki-ORF85	65,35	Thor-ORF63	73,74
74	lef-3	65798→67063	421	lef-3	54,36	lef-3	38,46	lef-3	51,8	Deki-ORF86	50,92	lef-3	55,26

75	Desmop	67058←69421	787	AcOrf-66	37,5	Desmop	64,58	desmop	37,62	Deki-ORF87	31,64	Thor-ORF61	36,6
76	DNA pol	69431→72454	1,007	Ac-DNA-pol	68,06	DNA polymerase	56,49	DNA pol	67,89	dnapol	68,27	DNA pol	67,7
77	*loob0*77	72980→73369	129	AcOrf-96	33,33					Deki-ORF89	36,15	pif-4	33,33
78	*loob0*78*	73445←73942	165										
79	*loob0*79	74119←74604	162	AcOrf-63	37,01			Mv-ORF47	33,77	Deki-ORF91	34,19	Thor-ORF58	36,18
80	lef-9	74707←76365	493	lef-9	82,52	lef-9	71,98	lef-9	83,13	lef-9	82,93	lef-9	83,13
81	fp-25k	76319→76954	211	fp-25k	75,12	fp-25k	68,68	fp-25k	80,22	fp-25k	76,22	fp-25k	73,17
82	ChaB-like	77113→77367	85	AcOrf-60	70	fp	60	Mv-ORF44	70	Deki-ORF94	70,49	ChaB-like	71,67
83	ChaB-like	77370→77522	50	AcOrf-59	78	ChaB-like	52	Mv-ORF43	70	Deki-ORF95	76	ChaB-like	76
84	*loob0*84	77598→77987	129										
85	*loob0*85	77973←78464	163	AcOrf-57	60,49	Anpe-ORF55	46,63			Deki-ORF96	62,96	Thor-ORF53	59,88
86	*loob0*86	78676←78981	102	AcOrf-56	46,53			Mv-ORF42	45,54	Deki-ORF97	45,76	Thor-ORF52	47,52
87	*loob0*87	78984←79202	72	AcOrf-55	52,63	Anpe-ORF54	52,63	Mv-ORF41	50	Deki-ORF98	52,63	Thor-ORF51	55
88	vp1054	79334←80437	367	AcOrf-54	70,33	vp1054	53,26	vp1054	69,23	vp1054	59,02	Thor-ORF50	71,15
89	lef-10	80295←80534	79	lef-10	62,82	lef-10	52,54	lef-10	58,97			lef-10	63,89
90	*loob0*90	80531←80962	143	AcOrf-53	78,68	Anpe-ORF51	58,09	Mv-ORF38	76,47	Deki-ORF100	73,57	Thor-ORF48	77,94
91	*loob0*91	81076→81606	177	AcOrf-52	47,78			Mv-ORF37	41,24	Deki-ORF101	44,69	Thor-ORF47	40,91
92	*loob0*92	81619←82611	331	AcOrf-51	38,94	Anpe-ORF50	28,48	Mv-ORF36	37,93	Deki-ORF102	32,17	Thor-ORF46	38,94
93	lef-8	82635→85298	887	lef-8	72,67	lef-8	64,61	lef-8	72,67	lef-8	69,45	lef-8	71,96
94	Pcna	85435→86253	273	pcna	50,2	Pcna	26,82			Deki-ORF104	47,43	pcna	48,44
95	*loob0*95	86299→86643	115	AcOrf-48	38,39	Etm	32,94						
96	Vef	86646→88940	764							vef-2	36,72		
97	*loob0*97	88846→89403	186										
98	ctl-1	89519←89680	53	ctl-1	60,38	ctl-1	60,38			ctl-1	58,49	ctl-1	60,38
99	bro-a	89729←90688	319	Ac-bro	42,47	bro-b	44,86			dk-bro-2	72,36	bro-b	61,18
100	he65**	90814←91473	220	he65	36,77	he65	29,18	he65	37,58			he65	28,95
101	odv-e66	91820←93916	698	Ac-odv-e66	79,02	odv-e66	69,57			odv-e66	33,05	odv-e66	76,49

102	*loob*102	93976←94365	130	AcOrf-44	52,89	Anpe-ORF44	36,04	Mv-ORF32	49,61	Deki-ORF107	52,63	Thor-ORF41	54,46
103	*loob*103	94352←94588	78	AcOrf-43	60,78	Anpe-ORF43	43,14	Mv-ORF31	59,57	Deki-ORF108	63,46		
104	lef-12	94616←95215	199	AcOrf-41	55	lef-12	36,9	lef-12	52,75	lef-12	43,85	lef-12	51,65
105	p47	95187→96422	402	Ac-p47	72,89	p47	64,27	p47	72,14	p47	69,31	p47	72,46
106	*loob*106	96554→97240	228	AcOrf-38	74,13	Anpe-ORF22	66,67	Mv-ORF27	72,6	Deki-ORF113	67,49	nudix protein	73,5
107	lef-11	97212→97574	120	Ac-lef11	47,06	lef-11	50	lef-11	49,45	lef-11	55,43	lef-11	50
108	39k/pp31	97564→98475	304	39k/pp31	55,23	39k/pp31	41,09	39k/pp31	55,04	39k/pp31	48,51	39k/pp31	53,43
109	v_ubi	98515←98751	79	v-ubi	94,74	v-ubi	89,47	v-ubi	89,47	v-ubi	92,21	v-ubi	92,31
110	*loob*110	98782→99414	211	AcOrf-34	50,94	Anpe-ORF26	45,86	Mv-ORF23	49,07	Deki-ORF117	46,67	Thor-ORF32	46,15
111	Fgf	99555→100106	183	fgf	47,19	Fgf	35,58			fgf	39,23	fgf	42,94
112	ctl-2	100097→100285	62	ctl-2	41,51	ctl-2	61,54			ctl-1	39,62	ctl-2	41,51
113	*loob*113*	100228←100617	130										
114	Sod	101182→101655	158	Ac-sod	75,51	Sod	79,05	sod	74,15	sod	69,74		
115	*loob*115	101752→103195	481	AcOrf-30	49,45	Anpe-ORF33	37,89	Mv-ORF21	46,59			Thor-ORF28	46,61
116	*loob*116	103234→103437	68	AcOrf-29	39,39	Anpe-ORF34	40,3	Mv-ORF20	55,22			Thor-ORF27	42,03
117	lef-6	103695←104354	219	lef-6	33,64	lef-6	36,17	lef-6	36,4	lef-6	41,09	lef-6	34,33
118	iap-1	104376←105308	310	iap-1	52,77	iap-1	44,81	iap-1	55,63	iap-1	49,7	iap-2	23,57
119	*loob*119	105320←105751	143	AcOrf-26	56,56	Anpe-ORF37	53,66	Mv-ORF17	52,46	Deki-ORF123	51,64	Thor-ORF24	56,25
120	Dbp	105828→106793	321	AcOrf-25	44,3	Dbp	40,83	dbp	44,37	dbp	62,89	dbp	44,01
121	Pkip	106907→107419	170	Ac-pkip	44,64	Pkip	37,5	pkip	41,92	Deki-ORF127	36,61	pkip	43,2
122	env-prot	107414←109483	689	Ac-env-prot	32,63	envelope	31,41	efp/ld130	31,04	Deki-ORF128	36,36	F	34,41
123	pif-2	109590←110735	381	AcOrf-22	77,17	pif-2	72,97	pif-2	77,43	pif-2	73,49	pif-2	76,38
124	Arif	110789→111808	339	AcOrf-21	33,33	arif-1	27,79	arif-1	30,71	arif-1	28,61	arif-1	32,69
125	*loob*125	111815←112129	104	AcOrf-19	42,2	Anpe-ORF18	39,8	Mv-ORF11	43,64	Deki-ORF132	49,02	Thor-ORF18	42,59
126	*loob*126	112228→113277	350	AcOrf-18	39,17	Anpe-ORF17	34,02	Mv-ORF10	38,76	Deki-ORF133	40,42	Thor-ORF17	39,64
127	*loob*127	113337←113972	211	AcOrf-17	56	Anpe-ORF16	41,71	Mv-ORF9	46,4			Thor-ORF16	50,72

128	bv/odv-e26	113941←114663	241	AcOrf-16	43,58	odv-e26	28,98	Mv-ORF8	40	bv/odv-e26	37,66	bv/odv-e26	43,53
129	Egt	114891←116435	514	Ac-egt	63,69	truncated EGT	48,65	egt	61,4	egt	62,17	egt	60,95
130	lef-1	116540→117244	234	lef-1	67,53	lef-1	50,65	lef-1	67,25	lef-1	64,35	lef-1	64,07
131	*loob*130	117237→118286	349	AcOrf-13	46,5	38.7K	31,05	38.7kDa	46,5	Deki-ORF137	37,58	Thor-ORF125	43,26
132	ptp-1	118304→118819	171	ptp-1	62,72	ptp-1	54,12	ptp-1	56,55			ptp-1	60,95
133	*loob*132	118906→119277	124	AcOrf-5	40,16	Anpe-ORF5	48,08	Mv-ORF125	39,68	Deki-ORF144	29,91	Thor-ORF5	35,77
134	lef-2	119258→119920	220	lef-2	65,24	lef-2	45,93	lef-2	62,86	lef-2	57,35	lef-2	62,38

**loob023* is not unique ORF and showed higher identity (49%) with Orf 009 from Anticarsia gemmatalis necleopolyhedrovirus (AgNV).

loob078 is not unique ORF and showed higher identity (43%) with Orf 101 from Pieris rapae granulovirus (PrGV).

loob113 is not unique ORF and showed higher identity (100%) with Orf 127 from Xestia c-nigrum granulovirus (XcGV).

** *loob100(he65)* showed higher identity (41%) with Orf 132 from Agrotis segetum granulovirus (AgseGV).

Table S2. List of 72 Baculovirus species used for phylogenetic analysis, concatenated amino acid sequence alignment of 37 baculovirus core genes.

Genera	Species	Abbreviation	Accession
Alphabaculovirus	*Adoxophyes honmai nucleopolyhedrovirus*	AdhoNPV	AP006270
Alphabaculovirus	*Adoxophyes orana nucleopolyhedrovirus*	AdorNPV	EU591746
Alphabaculovirus	*Agrotis ipsilon multiple nucleopolyhedrovirus* strain illinois	AgipMNPV	EU839994
Alphabaculovirus	*Agrotis segetum nucleopolyhedrovirus*	AgseNPV	DQ123841
Alphabaculovirus	*Antheraea pernyi nucleopolyhedrovirus* isolate L2	AnpeNPV-L2	EF207986
Alphabaculovirus	*Anticarsia gemmatalis nucleopolyhedrovirus*	AgMNPV	DQ813662
Alphabaculovirus	*Apocheima cinerarium nucleopolyhedrovirus*	ApciNPV	FJ914221
Alphabaculovirus	*Autographa californica nucleopolyhedrovirus* clone C6	AcMNPV-C6	L22858
Alphabaculovirus	*Bombyx mandarina nucleopolyhedrovirus* S2	BomaNPV-S2	JQ071499
Alphabaculovirus	*Bombyx mori nucleopolyhedrovirus* strain T3	BmNPV-T3	L33180
Alphabaculovirus	*Buzura suppressaria nucleopolyhedrovirus*	BusuNPV	KF611977
Alphabaculovirus	*Choristoneura fumiferana defective multiple nucleopolyhedrovirus*	CfDEFMNPV	AY327402
Alphabaculovirus	*Choristoneura fumiferana multiple nucleopolyhedrovirus*	CfMNPV	AF512031
Alphabaculovirus	*Choristoneura murinana nucleopolyhedrovirus*	ChmuNPV	KF894742
Alphabaculovirus	*Choristoneura occidentalis nucleopolyhedrovirus*	ChocNPV	KC961303
Alphabaculovirus	*Choristoneura rosaceana nucleopolyhedrovirus*	ChroNPV	KC961304
Alphabaculovirus	*Chrysodeixis chalcites nucleopolyhedrovirus*	ChchNPV	AY864330
Alphabaculovirus	*Clanis bilineata nucleopolyhedrovirus*	ClbiNPV	DQ504428
Alphabaculovirus	*Condylorrhiza vestigialis multiple nucleopolyhedrovirus*	CoveMNPV	KJ631623
Alphabaculovirus	*Dendrolimus kikuchii nucleopolyhedrovirus*	DekiNPV	JX193905
Alphabaculovirus	*Ectropis obliqua nucleopolyhedrovirus* strain A1	EcobNPV-A1	DQ837165
Alphabaculovirus	*Epiphyas postvittana nucleopolyhedrovirus*	EppoNPV	AY043265

Alphabaculovirus	*Euproctis pseudoconspersa nucleopolyhedrovirus*	EupsNPV	FJ227128
Alphabaculovirus	*Helicoverpa armigera multiple nucleopolyhedrovirus*	HaMNPV	EU730893
Alphabaculovirus	*Helicoverpa armigera nucleopolyhedrovirus* C1	HaNPV-C1	AF303045
Alphabaculovirus	*Helicoverpa zea single nucleopolyhedrovirus* USA	HzSNPV-USA	AF334030
Alphabaculovirus	*Hemileuca sp. nucleopolyhedrovirus*	HespNPV	KF158713
Alphabaculovirus	*Hyphantria cunea nucleopolyhedrovirus*	HycuNPV	AP009046
Alphabaculovirus	*Leucania separata nuclear polyhedrovirus* strain AH1	LeseNPV	AY394490
Alphabaculovirus	*Lymantria díspar multiple nucleopolyhedrovirus*	LdMNPV	AF081810
Alphabaculovirus	*Lymantria xylina multiple nucleopolyhedrovirus*	LyxyMNPV	GQ202541
Alphabaculovirus	*Mamestra brassicae multiple nucleopolyhedrovirus* strain Chb1	MbMNPV-CHb1	JX138237
Alphabaculovirus	*Mamestra configurata nucleopolyhedrovirus* B	MacoNPV-B	AY126275
Alphabaculovirus	*Mamestra configurata nucleopolyhedrovirus*-A strain 90/2	MacoNPV-A 90/2	U59461
Alphabaculovirus	*Maruca vitrata multiple nucleopolyhedrovirus*	MaviMNPV	EF125867
Alphabaculovirus	*Orgyia leucostigma nucleopolyhedrovirus* isolate CFS-77	OrleNPV	EU309041
Alphabaculovirus	*Orgyia pseudotsugata multiple nucleopolyhedrovirus*	OpMNPV	U75930
Alphabaculovirus	*Peridroma sp. nucleopolyhedrovirus*	PespNPV	KM009991
Alphabaculovirus	*Perigonia lusca single nucleopolyhedrovirus*	PeluSNPV	KM596836
Alphabaculovirus	*Philosamia cynthia ricini nucleopolyhedrovirus*	PhcyNPV	JX404026
Alphabaculovirus	*Plutella xylostella multiple nucleopolyhedrovirus* isolate CL3	PlxyMNPV	DQ457003
Alphabaculovirus	*Pseudoplusia includens single nucleopolyhedrovirus* IE	PsinSNPV	KJ631622
Alphabaculovirus	*Rachiplusia ou multiple nucleopolyhedrovirus*	RoMNPV	AY145471
Alphabaculovirus	*Spodoptera exigua nucleopolyhedrovirus*	SeMNPV	AF169823
Alphabaculovirus	*Spodoptera frugiperda multiple nucleopolyhedrovirus* isolate 19	SfMNPV-19	EU258200
Alphabaculovirus	*Spodoptera litoralis nucleopolyhedrovirus* isolate AN1956	SpliNPV-1956	JX454574

Alphabaculovirus	*Spodoptera litura nucleopolyhedrovirus* G2	SpliNPV-G2	AF325155
Alphabaculovirus	*Sucra jujuba nucleopolyhedrovirus*	SujuNPV	KJ676450
Alphabaculovirus	*Thysanoplusia orichalcea nucleopolyhedrovirus*	ThorNPV	JX467702
Alphabaculovirus	*Trichoplusia ni single nucleopolyhedrovirus*	TnSNPV	DQ017380
Betabaculovirus	*Adoxophyes orana granulovirus*	AdorGV	AF547984
Betabaculovirus	*Agrotis segetum granulovirus*-L1	AgseGV-L1	KC994902
Betabaculovirus	*Choristoneura occidentalis granulovirus*	ChocGV	DQ333351
Betabaculovirus	*Clostera anachoreta granulovirus*	ClanGV	HQ116624
Betabaculovirus	*Clostera anastomosis granulovirus*	CaLGV	KC179784
Betabaculovirus	*Cryptophlebia leucotreta granulovirus* isolate CV3	CrleGV	AY229987
Betabaculovirus	*Cydia pomonella granulovirus*	CpGV	U53466
Betabaculovirus	*Diatraea saccharalis granulovirus*	DisaGV	KP296186
Betabaculovirus	*Epinotia aporema granulovirus*	EpapGV	JN408834
Betabaculovirus	*Erinnyis ello granulovirus*	ErelGV	KJ406702
Betabaculovirus	*Helicoverpa armigera granulovirus*	HaGV	EU255577
Betabaculovirus	*Phthorimaea operculella granulovirus*	PhopGV	AF499596
Betabaculovirus	*Pieris rapae granulovirus* E3	PiraGV-E3	GU111736
Betabaculovirus	*Plutella xylostella granulovirus*	PlxyGV	AF270937
Betabaculovirus	*Pseudaletia unipuncta granulovirus*	PsunGV-Hawaiin	EU678671
Betabaculovirus	*Spodoptera frugiperda granulovirus*	SpfrGV	KM371112
Betabaculovirus	*Spodoptera litura granulovirus* isolate K1	SpliGV	DQ288858
Betabaculovirus	*Xestia c-nigrum granulovirus*	XcGV	AF162221
Gamabaculovirus	*Neodiprion abietis nucleopolyhedrovirus*	NeabNPV	DQ317692
Gamabaculovirus	*Neodiprion lecontei nucleopolyhedrovirus*	NeleNPV	AY349019

Gamabaculovirus	*Neodiprion sertifer nucleopolyhedrovirus*	NeseNPV	AY430810
Deltabaculovirus	*Culex nigripalpus nucleopolyhedrovirus*	CuniNPV	AF403738

Table S3. Restriction endonuclease fragment sizes (bp) of LoobMNPV DNA, from digestion performed *in* silico and from digestion performed with the same virus isolate from reference (Wolff et al., 2002)[6]

**PstI (*in silico*)**	**PstI [6]**	**EcoRI (i*n silico*)**	**EcoRI [6]**	**BamHI (*in silico*)**	**BamHI [6]**
24795	23430	13205	15870	43672	26330
19381	22930	13192	12500	21208	19380
17966	19370	9499	9150	13142	17130
17421	14130	9438	7350	9852	12300
17421	6530	7456	5500	9766	9300
17252	900	5724	5500	9056	8350
13036	500	5642	5150	8301	4430
6752		4253	3980	4615	
2141		4079	3200	411	
774		3450	3000		
505		3302	3000		
		3209	2770		
		3190	2570		
		2983	2400		
		2728	2270		
		2569	2130		
		2469	2070		
		2432	1670		
		2270	1450		
		2205	1350		
		2198	1200		
		2144	950		
		1742			
		1550			
		1467			
		1404			
		1364			
		1216			
		933			
		928			
		863			
		616			
		255			
		48			
